# Anaplastic, T-Cell, Non-Hodgkin's Lymphoma Presenting with Haematuria

**DOI:** 10.1100/tsw.2008.52

**Published:** 2008-03-25

**Authors:** C. Blick, S. Abdelhadi, D. Bailey, A. Muneer

**Affiliations:** ^1^ Department of Urology, Wycombe General Hospital, Buckinghamshire NHS Trust, UK; ^2^ Department of Pathology, Wycombe General Hospital, Buckinghamshire NHS Trust, UK

**Keywords:** bladder, lymphoma, anaplastic large-cell lymphoma, haematuria

## Abstract

Non-Hodgkin's lymphoma (NHL) represents about 3% of new cancer cases[1]. Bladder involvement has been found in approximately 3-13% of NHL patients when studied at postmortem[2]. Although accounting for only 0.2% of all primary bladder tumours, the majority of bladder lymphomas are B-cell lymphomas. T-cell lymphoma of the bladder is incredibly rare. We describe a case of anaplastic, T-cell lymphoma presenting with haematuria and loin pain, with unilateral upper tract obstruction.

## CASE SUMMARY

We describe the case of a 26-year-old man with an unusual presentation of lymphoma. He was referred to the urology department by his general practitioner with a 3-month history of back pain, macroscopic haematuria, weight loss, and night sweats. He was of Pakistani origin and as a child had received a full course of antituberculous treatment.

Initial investigations consisted of a renal tract ultrasound and intravenous pyelogram. The ultrasound scan revealed a thick-walled bladder with a heterogeneous mass arising from the bladder neck and prostate. A prominent left renal pelvis was also noted and a standing column of contrast in the left ureter to the vesicoureteric junction was seen at 1.5 h postcontrast. Rigid cystoscopy revealed a large, granulomatous, solid mass involving the prostate, bladder neck, trigone, and left ureteric orifice. The putative diagnosis at that time was tuberculosis (TB) of the bladder. The tumour was resected and histopathology revealed an aggressive, pleomorphic tumour. Immunohistochemistry was positive for ALK-1 (cytoplasmic and nuclear pattern), CD30, CD3, and CD4. Proliferation index with MIB-1 was high at 60% (Figs. 1–5). There were no stains for CD2, CD7, CD8, CD10, CD34, TdT, and CD79. A diagnosis of an ALK-positive, anaplastic, T-cell, non-Hodgkin's lymphoma was made. No genetic or molecular genetic studies were performed or T-cell clonality demonstrated. A staging CT scan showed extensive disease, with thoracic, axillary, retrocardiac, mediastinal, retroperitoneal, mesenteric, and pelvic lymphadenopathy. There were also multiple pulmonary nodules and a large lytic defect in the left innominate bone. Incidentally, multiple calcifications in his spleen and liver consistent with prior TB were also seen. He was commenced on the CHOP regime of chemotherapy (cyclophosphamide, doxorubicin, vincristine, and prednisone).

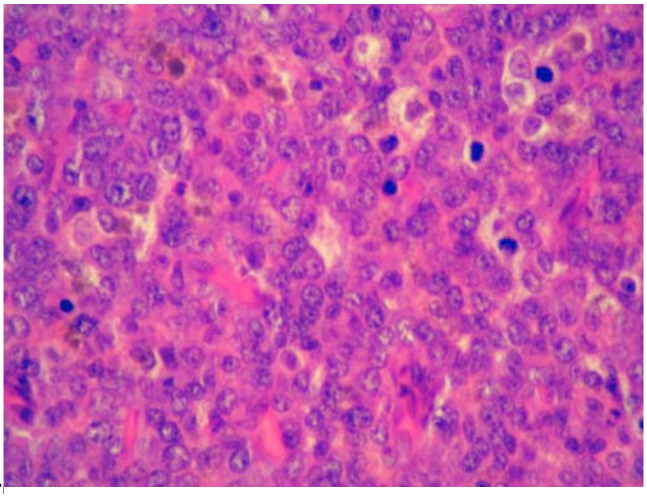

**FIGURE 1.** High power H&E of tumour.

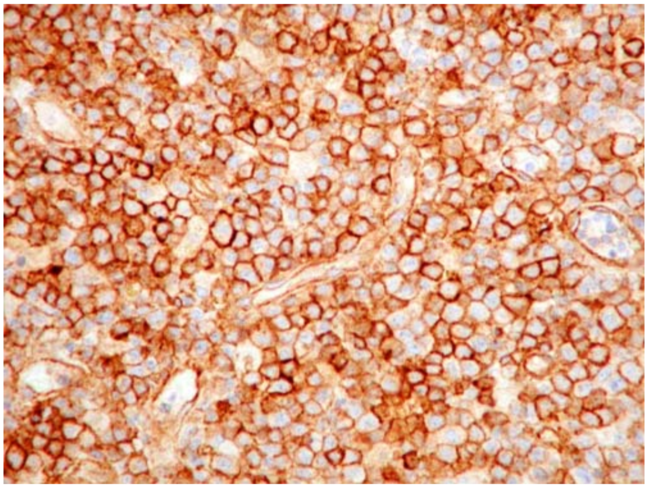

**FIGURE 2.** CD30 positivity.

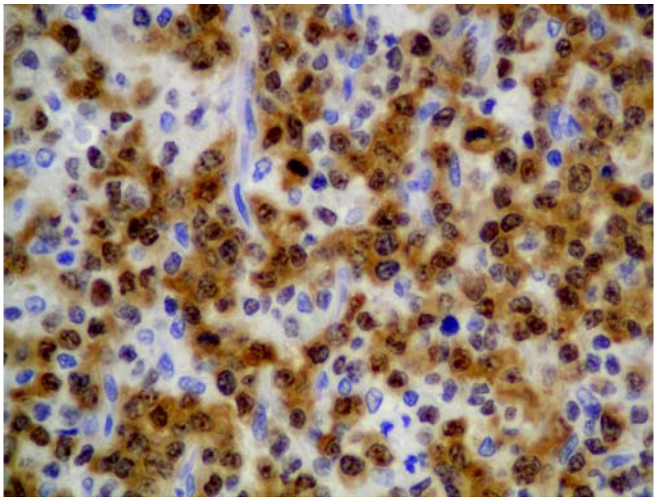

**FIGURE 3.** Alk 1.

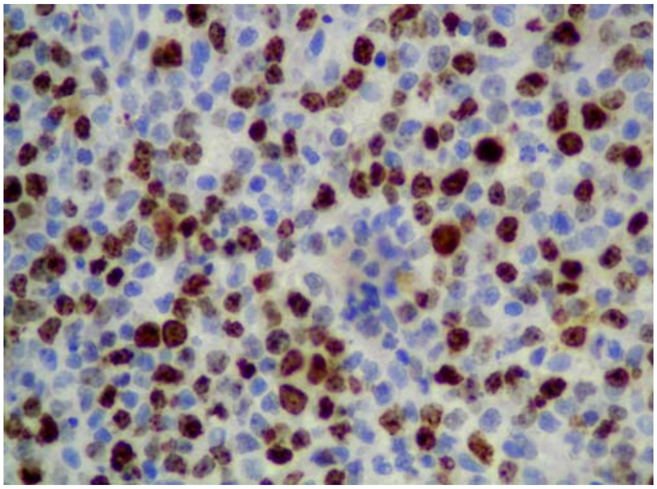

**FIGURE 4**. mib 1.

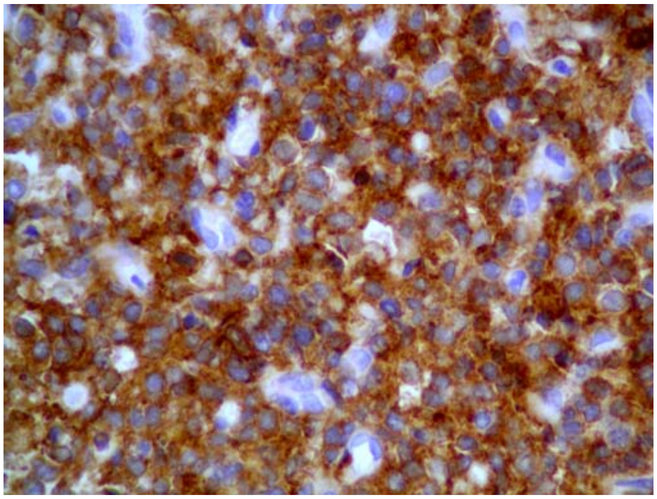

**FIGURE 5.** CD4.

## DISCUSSION

Urinary tract involvement in NHL is well documented, occurring in between 3 and 13% of NHL patients[3,4]. Bladder lymphomas are usually secondary deposits and can often be the presenting lesion of a previously unrecognised diffuse lymphomatous process[2]. Primary lymphoma of the bladder is very rare and nearly always B cell in origin[5]. There are only two reports of Hodgkin's lymphoma of the urinary bladder. The majority of bladder lymphomas are marginal zone lymphomas of mucosa-associated lymphoid tissue[5]. ALCL (anaplastic large cell lymphoma) involving the bladder is very unusual and there has only been one previous case presenting with haematuria[9]. ALCL is usually associated with cutaneous and extranodal involvement, young age at presentation, male predominance, and is not associated with urinary tract symptoms. Primary systemic ALCL is typically in an advanced stage at presentation and the disease is rapidly progressive. These patients have an increased incidence of bone marrow involvement (30%) and extranodal involvement, including skin (21%), bone (17%), soft tissues (17%), lung (11%), and liver (8%). Systemic symptoms are observed in 75% of patients, with fever the most common symptom. The malignant cells stain positive for the CD30 antigen, a very sensitive but nonspecific test result that is also positive in other lymphomas, including Hodgkin's disease( CD3 and alk positivity in this case excludes Hodgkin's). Most cases exhibit either T-cell or null phenotype, with frequent CD3 expression, cytotoxic protein expression, clonal T-cell receptor gene rearrangements by polymerase chain reaction (PCR), and lack of B-cell–associated markers. The primary systemic form, unlike the primary cutaneous form, generally stains positive for EMA and usually displays the t (2;5) translocation and the chimeric p80 protein with PCR and antibody studies[7]. The precise route that these tumours appear in the bladder is uncertain. The presence of lymphomas in the bladder may be related to either intrinsic foci or haematogenous spread causing tumour with multiple foci[6,8]. This case is unique, as T-cell lymphoma presenting with haematuria and upper tract obstruction has not previously been described. Lymphoma should be considered under differential diagnosis of bladder tumours, particularly if there are systemic symptoms or atypical features concerning the patient demographics or clinical history.
